# Editor’s introduction to this issue (G&I 17:3, 2019)

**DOI:** 10.5808/GI.2019.17.3.e22

**Published:** 2019-09-27

**Authors:** Taesung Park

**Affiliations:** Department of Statistics, Seoul National University, Seoul 08826, Korea

In this issue, there are 12 articles: two Review Articles, one short Mini Review, five Original Articles, two Research Communications, and two Opinion articles.

The first review by Park and Chung (The Catholic University of Korea, Korea) is about the role of neoantigens derived from alternative splicing and RNA modification. Neoantigens expressed from genes with mutations acquired during carcinogenesis may be tumor-specific and thus can be potential targets for personalized neoantigen-based immunotherapy. The authors summarized recent works on the large-scale screening of neoantigens produced by alternative splicing and RNA editing. The second review by Lee (National Forensic Service, Korea) focused on early-life nutrition which is known to be a major contributor to the permanent program change of organ structure and function toward the development of diseases. Especially, the author studied the relationship between gut microbiome and nutrients on development of disease and how microbiome modulation regulates epigenetic changes and influences human health. The third review by Kim et al. (Ewha Womans University, Korea) is about *Genomics & Informatics* journal. This short review provided a historical review by providing statistics of *Genomics & Informatics* regarding publication types, word clouds, and the most studied genes.

This issue contains five Original Articles. First, Kim et al. (Ehwa Womans University, Korea) presented a bioinformatics tool, FusionScan for predicting fusion transcripts from RNA-sequencing (RNA-Seq) data. Note that fusion gene has a high potential as carcinogenic drivers. Thus, its identification is of great interest in cancer research area. FusionScan seems to be a reliable, efficient and convenient program for detecting fusion transcripts that meet the requirements in the clinical and experimental community. Kim et al. (KRIBB, Korea) provided an optimization process of a microarray for fission yeast. It is well known that bar-code (tag) microarrays of yeast gene-deletion collections allow the systematic identification of genes required for growth in any condition of interest. Thus, this optimized microarray is expected to be a powerful analytical platform for elucidating currently unknown gene functions.

Lee et al. (Chonbuk National University, Korea) presented an interesting novel concept, transcript capacity (TC) referring to the capacity that a transcript exerts in a cell as enzyme or protein function after translation. TC can be estimated through an in silico method using the data from the effect sizes derived from genome-wide association studies and transcription level in RNA-seq to estimate TC. While TC needs some further investigation, it seems to be a totally new concept. The next article by Jang et al. (Seoul National University College of Medicine, Korea) investigated human leukocyte antigen (HLA) difference between the two control datasets of The Wellcome Trust Case Control Consortium (WTCCC). They showed that the genomic contents are not significantly different between control suggesting that the combined controls can be used as controls for HLA fine-mapping analysis based on HLA imputation.

The final Research Article is by Park et al. (Kangwon National University, Korea). They provided *de novo* transcriptome sequencing and gene expression profiling data with/without B-chromosome plants of *Lilium amabile* along with the functional enrichment analysis. This study is expected to provide insight into transcriptomic changes and evolution of plant B chromosomes in the Korean lily.

In this issue, there are two Research Communication articles. Jeon et al. (KRIBB, Korea) investigated the performance of new sequencer MGISEQ-2000 MGI Tech by comparing its performance with that of HiSeq 4000 from Illumina. They tested whether or not a new MGISEQ-2000 sequencer delivers the high-quality sequence data. Data produced from the MGISEQ-2000 and HiSeq 4000 had high concordance rate. Thus, they concluded that the performance of MGISEQ-2000 is comparable to HiSeq 4000 and that MGISEQ-2000 can be a useful platform for sequencing. This comparison result will be quite helpful to the researchers. Park and Nam (Gachon University, Korea) presented a neuroblastoma stage classification by using deep learning of prediction from gene expression data. Since neuroblastoma is known to be one of lethal cancer types in early childhood, its early diagnosis is critical. This deep learning model could play an important role in neuroblastoma stage classification.

Finally, two Opinion articles are about direct to consumer (DTC) genetic testing by Oh (Kyung Hee University, Korea) and by Kim (Sungkyunkwan University, Korea). Direct-to-Consumer (DTC) genetic testing is a worldwide controversial issue. Recently, several biotech companies in Korea started DTC genetic testing service for a few health related phenotypes. The opinions of the two authors’ are timely. It would be important for the consumers to be aware of what is the DTC genetic testing, what are the advantages and disadvantages of DTC genetic testing, how to make up for the shortcomings of the DTC genetic testing, what are their scientific and medical issues, and how to cope with the legal issues and principle of transparency.

